# Pityriasis rosea after Moderna mRNA‐1273 vaccine: A case series

**DOI:** 10.1111/dth.15225

**Published:** 2021-12-01

**Authors:** Fabrizio Martora, Gabriella Fabbrocini, Claudio Marasca

**Affiliations:** ^1^ Dermatology Unit, Department of Clinical Medicine and Surgery University of Naples Federico II Naples Italy

Dear Editor,

The SARS‐CoV‐2 pandemic plagued the world over the course of the year. Several vaccines have been created to counteract the morbidity and mortality associated with COVID‐19 and stop viral transmission, including the Moderna vaccine (Mrna‐1273).The mRNA‐1273 vaccine is a lipid nanoparticle–encapsulated mRNA‐based vaccine that encodes the prefusion stabilized full‐length spike protein of the severe acute respiratory syndrome coronavirus 2 (SARS‐CoV‐2), the virus that causes Covid‐19^1^ The adverse reactions which occur with messenger RNA (mRNA) COVID‐19 vaccines mostly appear within the first 30 min after the vaccination and can likely be interpreted as immunoglobulin E‐mediated hypersensitivity. Delayed cutaneous adverse events reported, other than injection site inflammation, are rare. As a growing percentage of the population becomes vaccinated, a variety of delayed cutaneous reactions are also beginning to be reported. To date in literature are reported few cases of delayed cutaneous reactions, among these a delayed urticaria and an erythema multiforme like eruption, eczematous eruptions, generalized pruritic morbilliform, urticarial vasculitis and leukocytoclastic vasculitis. Both for immediate and delayed reactions it could be useful to perform skin testing to demonstrate the culprit role of vaccine. Few cases of bullous pemphigoid up to more unusual reactions, such as erythromelalgia, pernio/chilblains, filling reactions and pityriasis rosea‐like rashes have also been reported.^2^, ^3^, ^4^, ^5^

Here we report three cases of patients who presented with pityriasis rosea after the first dose of the vaccine (Moderna).

In the first case, a 46‐year‐old woman presented to our dermatology department with erythematous and desquamative lesions localized mainly to the trunk interrupting at the root of the legs. During history collection, it was revealed that the patient had received the first dose of Moderna mRNA‐1273 vaccine 6 days before the rash. In the second case, a 49‐year‐old man presented with the same manifestations on the trunk 7 days after vaccination. Finally, a 24‐year‐old woman showed the same manifestations placed on the trunk 11 days after vaccination. None of the three cases mentioned had other symptoms associated to the rash, except for patient 1 who, suffering from hidradenitis suppurativa, presented a worsening of HS manifestations. On dermatological physical examination, eiritematous desquamative lesions were found, slightly itchy, distributed all over the trunk following the typical “Christmas tree” pattern of the pathology (Figure 1).

**FIGURE 1:**
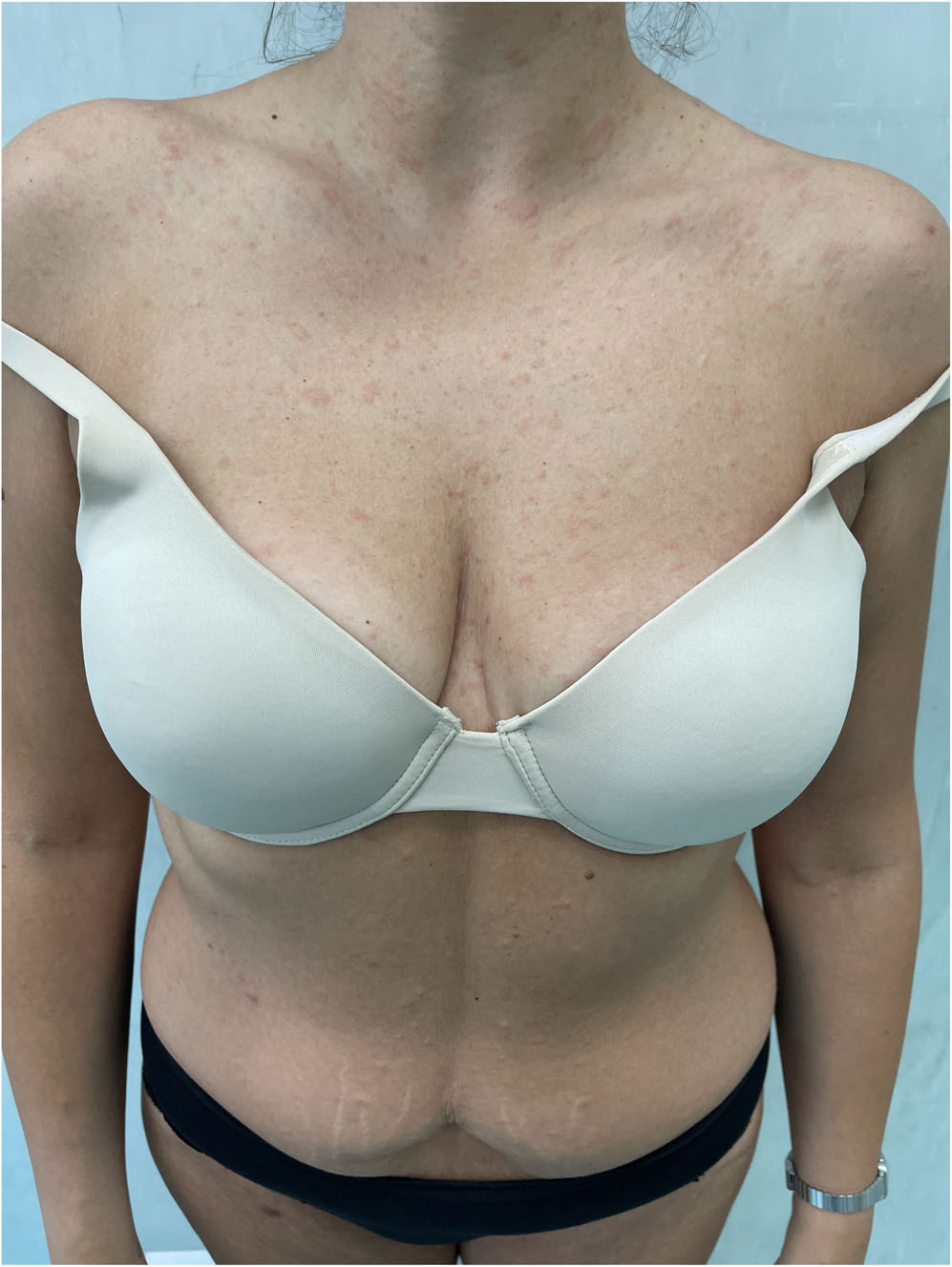
Diffuse erythematous desquamative lesions from the neck region to the root of the legs

Based on the clinical history and physical examination, a diagnosis of pityriasis rosea was made and, according to the guidelines, topical therapy with corticosteroids and oral therapy with antihistamines was prescribed like with resolution of PR manifestations.^6^

We want to emphasize that all three patients have normally undergone the second dose of the vaccine because these manifestations are absolutely not a contraindication to the second dose.

There are dates in the literature that have demonstrated that SARS‐CoV‐2 infection may have played a role in the reactivation of HHV‐6, ‐7, and EBV and, consequently, caused skin manifestations typical of pityriasis rosea,^7^ probably causes an immunosuppressive state secondary to a decrease in the amount of T‐lymphocytes. This immunosuppressive state would explain the reactivation of many viruses to which can follow the appearance of skin manifestations such as pityriasis rosea.^8^

Several vaccines have rarely been reported to induce pityriasis rosea, including vaccinations for smallpox, tuberculosis, influenza, papillomavirus, polio, tetanus, diphtheria, pneumococcal, diphtheria‐pertussis‐tetanus, hepatitis B, and yellow fever.^9^ Our article wants to put a point of reflection on the correlation between the vaccine mRNA 1273 and PR. In view of the mass vaccination on a global scale, we believe it is fundamental to report this case series since very few cases of this type have been described in the literature, we believe at the same time that there is still much to discover about the physiopathological mechanisms underlying the dermatological manifestations after the mRNA 1273 vaccine. Nevertheless, further studies are needed to confirm our hypothesis (written informed consent: Patients has given the consent for the use of image and publication of their case details).

## CONFLICT OF INTEREST

The authors declare no conflicts of interest.

## Data Availability

The data that support the findings of this study are available from the corresponding author upon reasonable request.
